# Significance of liver resection for intermediate stage hepatocellular carcinoma according to subclassification

**DOI:** 10.1186/s12885-021-08421-3

**Published:** 2021-06-05

**Authors:** Masateru Yamamoto, Tsuyoshi Kobayashi, Masakazu Hashimoto, Shintaro Kuroda, Tomokazu Kawaoka, Hiroshi Aikata, Kazuaki Chayama, Hideki Ohdan

**Affiliations:** 1 Department of Gastroenterological and Transplant Surgery, Graduate School of Biomedical and Health Sciences, Hiroshima University, 1-2-3, Kasumi, Minami-ku, Hiroshima City, Hiroshima, 734-8551 Japan; 2 Research Center for Hepatology and Gastroenterology, Hiroshima University, Hiroshima, Japan; 3 Department of Gastroenterology and Metabolism, Graduate School of Biomedical and Health Sciences, Hiroshima University, Hiroshima, Japan

**Keywords:** BCLC staging system, Hepatocellular carcinoma, Intermediate stage, Kinki criteria, Liver resection

## Abstract

**Background:**

Patients diagnosed with Barcelona Clinic Liver Cancer (BCLC) intermediate stage hepatocellular carcinoma (HCC) encompass a broad clinical population. Kinki criteria subclassifications have been proposed to better predict prognoses and determine appropriate treatment strategies for these patients. This study validated the prognostic significance within the Kinki criteria substages and analyzed the role of liver resection in patients with intermediate stage HCC.

**Methods:**

Patients with intermediate stage HCC (*n* = 378) were retrospectively subclassified according to the Kinki criteria (B1, *n* = 123; B2, *n* = 225; and B3, *n* = 30). We analyzed the overall survival (OS) and treatment methods.

**Results:**

The OS was significantly different between adjacent substages. Patients in substage B1 who underwent liver resection had a significantly better prognosis than those who did not, even after propensity score matching (PSM). Patients in substage B2 who underwent liver resection had a significantly better prognosis than those who did not; however, there was no difference after PSM. There was no difference in prognosis based on treatments among patients in substage B3.

**Conclusions:**

The Kinki criteria clearly stratify patients with intermediate stage HCC by prognosis. For substage B1 HCC patients, liver resection provides a better prognosis than other treatment modalities. In patients with substage B2 and B3, an alternative approach is required.

**Supplementary Information:**

The online version contains supplementary material available at 10.1186/s12885-021-08421-3.

## Introduction

Different staging systems have been proposed for hepatocellular carcinoma (HCC) [1–4]. The Barcelona Clinic Liver Cancer (BCLC) staging system has gained widespread acceptance due to its inclusion of patient-related factors, tumor extent, and liver function, as well as the algorithmic component for treatment recommendations [1, 5, 6]. However, the range of patients belonging to the intermediate stage is wide. The intermediate stage includes patients with clinical findings from two tumor nodules over 3 cm in diameter to extensive bilobular infiltration, and from the uncompromised stage to the cirrhotic stage (Child-Pugh B with nine points and severely impaired liver function). Despite its wide range of patients, the BCLC staging system indicates one treatment for BCLC intermediate stage patients with HCC: transarterial chemoembolization (TACE). In practice, various treatments are performed due to the heterogeneity of liver function and tumor burden in patients in intermediate stage, and the survival rate differs depending on the treatment method [7–9]. Therefore, treatment strategies should be adapted according to a subdivision of this heterogeneous patient population.

Bolondi et al. [10] proposed a subclassification of BCLC intermediate stage patients with HCC due to the heterogeneity of this group. This substaging system incorporates an assessment of liver function using the Child–Pugh score and that of tumor burden using beyond the Milan criteria and within the up-to-7 criteria [11]. While TACE is the only recommended treatment for BCLC intermediate stage HCC, individual treatment recommendations are associated with these substages. However, the substages are complicated, and no treatment option is recommended for substage B3. In addition, liver transplantation is recommended as an alternative treatment option for patients in substage B1; however, this treatment option is unrealistic, as it is limited by current local regulations and organ scarcity. Therefore, Kudo et al. [12] proposed the Kinki criteria that modified the substaging system to include proposed treatment strategies. The Kinki criteria are easier to apply than Bolondi’s substaging system and include more detailed treatment recommendations for each substage. For example, the treatment recommendations for substage B1 include resection and hepatic artery infusion chemotherapy (HAIC) is recommended for patients with multiple HCCs that exceed the up-to-7 criteria. For patients with impaired liver function, embolization with drug-eluting beads is recommended.

Liver resection is not recommended in patients with the BCLC intermediate stage treatment algorithm. However, for patients with preoperatively well-preserved liver function and postoperatively sufficient functional liver remnant, liver resection is performed in selected patients with multiple or even macrovascular invasive HCC [13]. The role of surgery has been extended beyond the BCLC algorithm, with improved survival outcomes reported in patients with intermediate and advanced stage HCC compared to patients receiving other treatments [14, 15]. Therefore, there is a large gap between clinical guidelines and real-world practice, and liver resection may be beneficial for patients with BCLC intermediate or advanced stage.

It is important to subdivide the heterogeneous group of patients in BCLC intermediate stage and provide guidance based on liver function, tumor burden, and patient factors for therapeutic decisions. Therefore, we investigated the survival benefit of liver resection over non-surgical treatment in HCC patients with BCLC intermediate stage HCC subclassification.

## Methods

### Study design

A total of 2387 patients diagnosed with HCC who were treated at Hiroshima University between 2000 and 2016 were included in this retrospective cohort study. HCC was diagnosed either by radiological or pathological findings. Dynamic computed tomography (CT) or dynamic magnetic resonance imaging (MRI) showed hyperattenuation in the arterial phase and hypoattenuation in the portal venous or equilibrium phases in liver lesions. Elevated serum levels of α-fetoprotein (AFP) and des-γ-carboxy prothrombin (DCP) were also considered. When performing treatment other than resection, a biopsy was performed before treatment to improve the certainty of diagnosis. The eligibility criteria were a BCLC intermediate stage classification [1] (defined as two to three nodules ≥3 cm or four or more nodules), an Eastern Cooperative Oncology Group (ECOG) performance status of 0, and a Child-Pugh grade of A or B. Patients with recurrence, distant metastasis, or simultaneous malignancies were excluded. Overall, 378 patients met these criteria and were enrolled. The determination of the substage according to the Kinki criteria [12] was followed; Kinki criteria are divided into three substages based on the Child-Pugh score (5–7 or 8–9) and beyond the Milan criteria and within the up-to-7 criteria (in or out). The substage B1 includes patients with Child-Pugh scores of 5–7 points and beyond the Milan criteria and within the up-to-7 criteria in; the substage B2 comprises patients with Child-Pugh scores of 5–7 points and beyond the Milan criteria and within the up-to-7 criteria out; substage B3a and B3b are composed of patients with Child-Pugh scores of 8–9 points and beyond the Milan criteria and within the up-to-7 criteria in and out, respectively. Various treatment modalities were presented to patients throughout all substages and were selected by the patients themselves. Patients were divided into the resection and non-resection groups. The demographic data were collected from the hospital database.

### Treatment procedure

#### Liver resection

The appropriate liver resection procedure was selected after assessing the liver function, patient factors, and tumor factors. Liver function was assessed based on the general rules for the clinical and pathological study of primary liver cancer [16]. A standard liver resection procedure was performed in this study, as previously described [17, 18]. Hepatic blood flow was blocked using the Pringle technique. The number of tumors and tumor size, location, and infiltration into surrounding organs were assessed using intraoperative ultrasonography.

#### Transarterial chemoembolization

The TACE procedure was performed as previously described [19]. In brief, a microcatheter was inserted through the femoral artery into a segment or sub-segment of the hepatic nutrient artery containing the target tumor. Cisplatin (7–70 mg/body at a concentration of 10 mg/mL; Randa, Nippon Kayaku, Tokyo, Japan) mixed with iodized oil (Laboratoire Guerbet, Villepinte, France) was injected via the arterial branches. Embolization was induced using a small amount of gelatin sponge particles until the flow of the feeding artery was markedly reduced.

#### Hepatic arterial infusion chemotherapy

Arterial infusions of anticancer agents and the placement of infusion ports were performed as previously described [20]. The patients received a combination of low-dose cisplatin and 5-Fluorouracil. Cisplatin (20 mg/m^2^ per day; Nippon Kayaku, Tokyo, Japan) was administered intra-arterially on days 1 and 8. 5-Fluorouracil (300 mg/m^2^ per/day; Kyowa Hakko, Tokyo, Japan) was administered continuously on days 1–5 and 8–12 via a mechanical infusion pump.

#### Sorafenib

Sorafenib (standard dose: 800 mg/day) was used for the treatment of patients with HCC from 2009 [21]. When adverse events occurred, the dose was tapered to 400 mg/day or the medication was withheld, depending on the severity of the adverse events.

#### Follow-up

All patients received a routine examination at a follow-up one month after hospital discharge, then every three months in the first year. Follow-up visits were every six months thereafter. Patients were screened for recurrence or metastases via tumor marker measurements, abdominal ultrasonography, CT, and MRI.

#### Statistical analysis

Overall survival (OS) was defined as the duration from the date of treatment or, in the case of best supportive care (BSC) the date of diagnosis, to the date of the last follow-up visit or death. Continuous variables are presented as median (range), and categorical variables are presented as numbers (frequencies). Continuous variables were evaluated using the Wilcoxon Rank Sums tests and categorical variables were evaluated using the Fisher’s exact test. The cut-off points for continuous variables were determined by receiver operating characteristic curve analysis with median overall survival as the outcome. Survival curves were depicted using the Kaplan-Meier method, and a comparison of OS was analyzed using the log-rank test. Variables that were significant on the univariate analysis were entered into a multivariate Cox regression analysis. To minimize the selection bias, propensity score matching (PSM) was used with multiple logistic regression according to the patients’ baseline characteristics. The following covariates, which have been considered to affect prognosis in other studies, were adjusted; age, sex, hepatitis B and C viruses, serum total bilirubin concentration, albumin levels, prothrombin activity, platelet count, indocyanine green retention rate at 15 min (ICGR15), serum AFP levels, DCP levels, Tumor number, and tumor size [17, 22, 23]. One-to-one matching was performed using nearest neighbor method with a caliper of 0.20. Cohen’s D was calculated to confirm the matching balance. Statistical analyses were performed using JMP Pro software (version 14; SAS Institute, Cary, NC, USA). Statistical significance was set at *p* < 0.05.

## Results

### Baseline characteristics

**Fig. 1:**
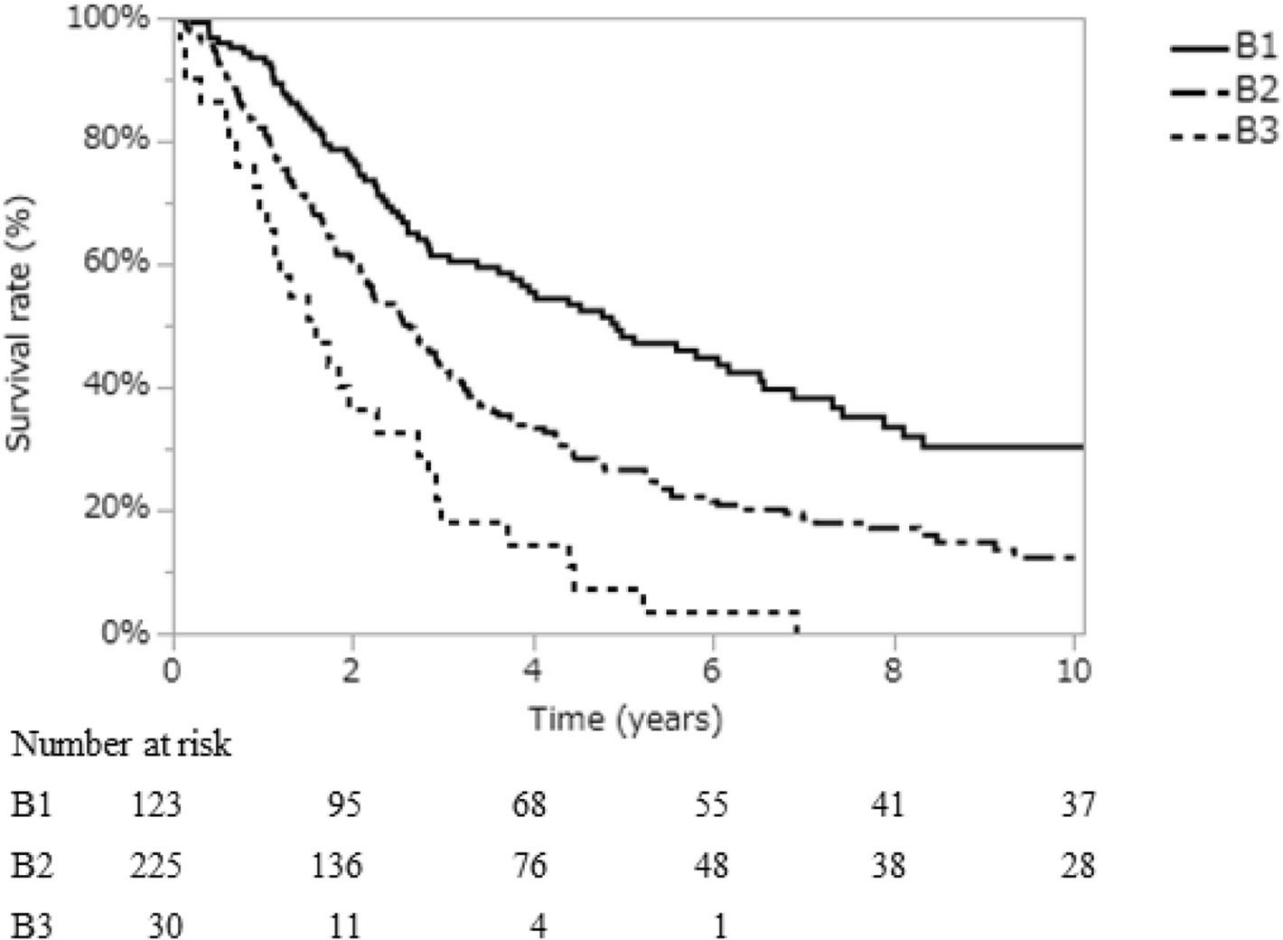
Overall survival by substage in BCLC stage.B. Survival curves differed significantly in adjacent substages (B1 vs. B2, *p* < 0.001; and B2 vs. B3, p < 0.001)

Among the 378 patients who were included in this study, 123 (32.5%) were classified as substage B1, 225 (59.5%) as substage B2, and 30 (8.0%) as substage B3. The median age of patients was 70 years (range, 26–93 years), and the majority of patients (301/378; 79.6%) were male. Hepatitis B virus surface antigen was positive in 62 patients (16.4%), while 198 patients (52.7%) were positive for the hepatitis C virus antibody (Table 1). A total of 302 (79.9%) were classified as Child-Pugh grade A. The median OS was 2.8 years (range, 0.1–16.4 years). The OS was clearly stratified between patients in the substages B1, B2, and B3 groups. The median OS was 4.9 years (range 0.2–16.3 years) in the substage B1 group, 2.6 years (range, 0.1–16.4 years) in the substage B2 group, and 1.5 years (range, 0.1–6.9 years) in the substage B3 group. The OS differed significantly in adjacent substages (B1 vs. B2, *p* < 0.001; and B2 vs. B3, p < 0.001; Fig. 1). The cancer specific survival (CSS) is shown in Figure S1. Similarly, the CSS differed significantly in adjacent substages (B1 vs. B2, p < 0.001; and B2 vs. B3, p < 0.001).

**Table 1:** Baseline characteristics

	B1 *N* = 123 (32.5)	B2 *N* = 225 (59.5)	B3 *N* = 30 (8.0)
Age (years)	70 (45–93)	70 (26–93)	68 (50–86)
Sex			
Male	104 (84.5)	175 (77.8)	22 (73.3)
Female	19 (15.5)	50 (22.2)	8 (26.7)
HBV positive	16 (13.1)	40 (17.8)	6 (20.7)
HCV positive	81 (65.8)	105 (46.7)	12 (42.9)
Plt (× 104/mm3)	11.5 (3.3–189)	14.3 (3.7–222)	10.7 (4.9–94.3)
PT (%)	83 (24–112)	86 (14–139)	64 (37–101)
T-Bil (mg/dL)	0.9 (0.3–2.8)	0.8 (0.3–2.5)	1.6 (0.3–3.2)
AST (IU/L)	41 (17–568)	50 (13–432)	45 (16–296)
ALT (IU/L)	37 (11–834)	41 (10–388)	33 (11–204)
Alb (g/dL)	3.9 (2.8–5.2)	3.8 (0.2–5.1)	2.7 (2.0–3.5)
ICGR15 (%)	20 (2.6–79.2)	16.5 (1.0–79.1)	39.0 (6.6–76.9)
AFP (ng/mL)	19.8 (0.5–8390)	34.3 (0.5–449,860)	60.8 (0.5–25,910)
DCP (mAU/mL)	79.5 (10–32,365)	345.5 (1.6–529,200)	333.5 (10–4,673,500)
Child-Pugh			
A	107 (87.0)	195 (86.7)	0 (0)
B	16 (13.0)	30 (13.3)	30 (100.0)
Up to seven			
In	123 (100)	–	10 (33.3)
Out	–	225 (100)	20 (66.7)
Tumor number	2 (2–5)	5 (2–20)	3 (2–15)
Tumor size (mm)	35 (7–50)	50 (8–355)	50 (13–200)
Treatment modalities			
Resection	70 (56.9)	88 (39.1)	6 (20.0)
TACE	49 (39.8)	125 (55.6)	18 (60.0)
Ablation	4 (3.0)	–	–
HAIC	–	9 (4.0)	2 (6.7)
Systemic chemotherapy	–	3 (1.3)	–
Transplantation	–	–	1 (0.3)
BSC	–	–	3 (10.0)

*AFP* alpha-fetoprotein, Alb albumin, *AST* asparate aminotransferase, *ALT* alanine aminotransferase, *BSC* best supportive care, *DCP* des-gamma-carboxyprothorombin, *HAIC* hepatic arterial infusion chemotherapy, *HBV* hepatitis B virus, *HCV* hepatitis C virus, *ICGR15* indocyanine green retention rate at 15 min, *Plt* platelet count, *PT* prothrombin time, *TACE* transarterial chemoembolization, *T. Bil* total bilirubin

### Substage B1

**Fig. 2:**
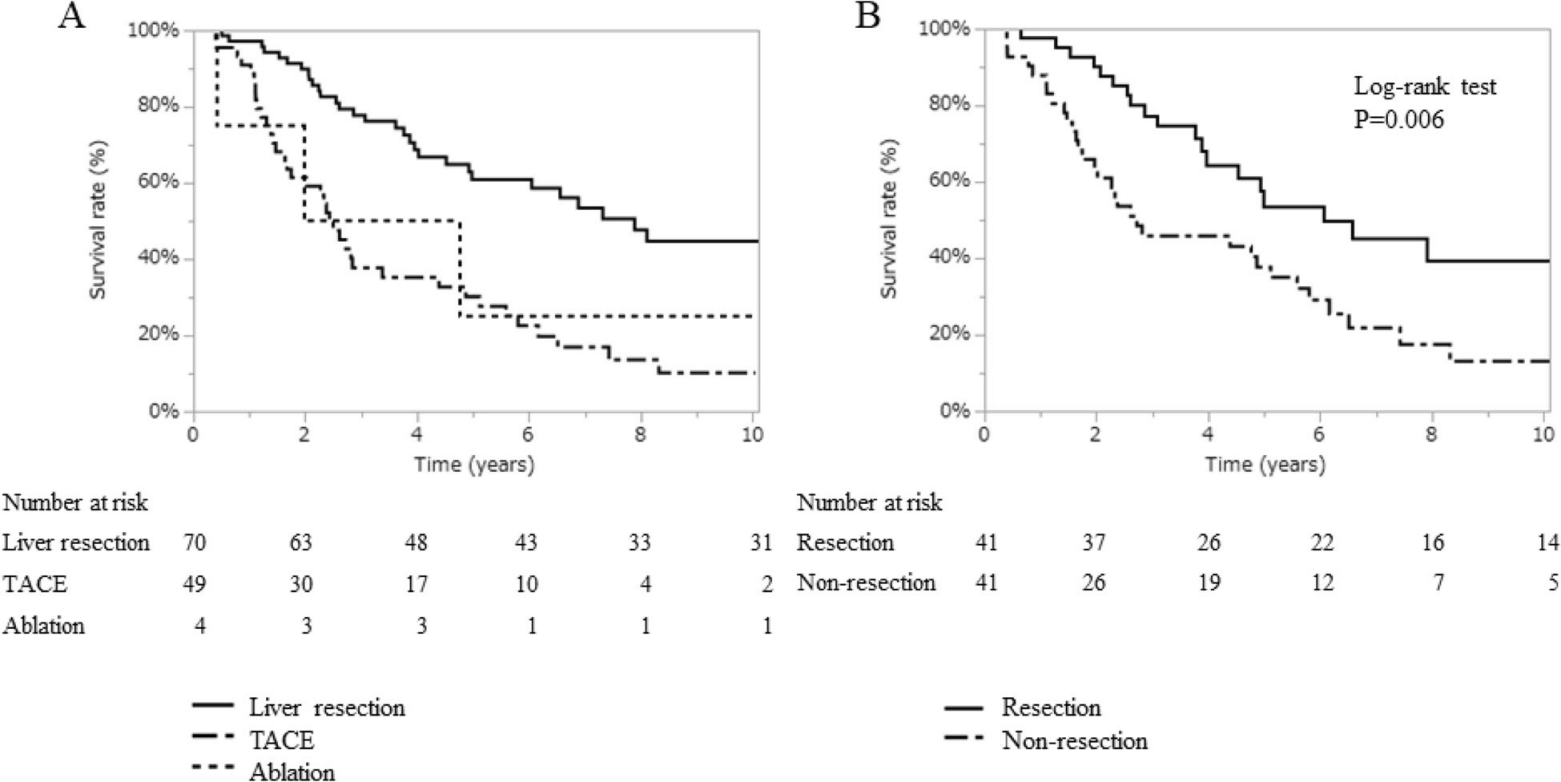
Overall survival in substage B1. **A**. Survival curves showing the prognostic impact by treatment. **B**. Survival curves between resection and non-resection in substage B1 after propensity score matching. TACE: transarterial chemoembolization

The median OS was 7.9 years (range 0.5–16.3 years) in the liver resection group, 2.4 years (range, 0.2–10.0 years) in the TACE group, and 2.0 years (range, 0.4–10.8 years) in the ablation group (Fig. 2A). Table S1 shows the demographics of patients in substage B1 by treatment types. There was a tendency toward younger patients and Child-Pugh grade A in the resection group. The results of the univariate and multivariate analyses of OS are listed in Table S2. Independent factors for favorable OS included AFP ≤37 ng/mL and liver resection. Patients in substage B1 were divided into two groups: the resection group and the non-resection group (Table 2). Total bilirubin and ICGR15 were lower in the resection group. To minimize the selection bias of these liver function tests, PSM was performed and resulted in comparable patient demographics (Table S3). Figures S2A and C show the distributions of propensity scores before and after matching. The OS of the resection group remained significantly higher than that of the non-resection group after PSM (Fig. 2B). The results of the univariate and multivariate analyses of OS are listed in Table S4. Independent factors for favorable OS were similar before and after PSM. CSS for each treatment before PSM is shown in Figure S3A, and CSS in the resection and non-resection groups after PSM is shown in Figure S3B.

**Table 2:** Patient background in substage B1

	Non-resection *N* = 53 (43.1)	Resection *N* = 70 (56.9)	*P*-value	Cohen’s D
Age (years)	70 (51–93)	68 (45–87)	0.121	0.333
Sex			0.607	0.165
Male	43 (81.1)	61 (87.1)		
Female	10 (18.9)	9 (12.9)		
HBV positive	6 (11.3)	10 (14.3)	0.788	0.089
HCV positive	31 (58.5)	50 (71.4)	0.181	0.273
Plt (×104/mm3)	10.5 (3.6–25.5)	12.5 (3.3–189)	0.168	0.214
PT (%)	82 (36–112)	84 (24–112)	0.064	0.312
T-Bil (mg/dL)	0.9 (0.4–2.8)	0.8 (0.3–2.2)	0.013	0.536
AST (IU/L)	42 (17–568)	42 (17–148)	0.513	0.283
ALT (IU/L)	35 (11–834)	39 (11–165)	0.732	0.202
Alb (g/dL)	3.9 (2.8–4.7)	3.9 (2.9–5.2)	0.865	0.102
ICGR15 (%)	23.1 (4.0–66.7)	17.4 (2.6–79.2)	0.008	0.492
AFP (ng/mL)	28.7 (0.5–4172)	14.1 (1.4–8390)	0.211	0.545
DCP (mAU/mL)	74 (10–17,093)	96 (10–32,365)	0.939	0.218
Child-Pugh	43/10 (81.1/18.9)	64/6 (91.4/8.6)	0.106	0.302
A	43 (81.1)	64 (91.4)		
B	10 (18.9)	6 (8.6)		
Tumor number	2 (2–5)	2 (2–4)	0.116	0.309
Tumor size (mm)	35 (7–46)	36 (10–50)	0.054	0.335

*AFP* alpha-fetoprotein, *Alb* albumin, *AST* asparate aminotransferase, *ALT* alanine aminotransferase, *DCP* des-gamma-carboxyprothorombin, *HBV* hepatitis B virus, *HCV* hepatitis C virus, *ICGR15* indocyanine green retention rate at 15 min, *Plt* platelet count, *PT* prothrombin time, *T. Bil* total bilirubin

### Substage B2

**Fig. 3:**
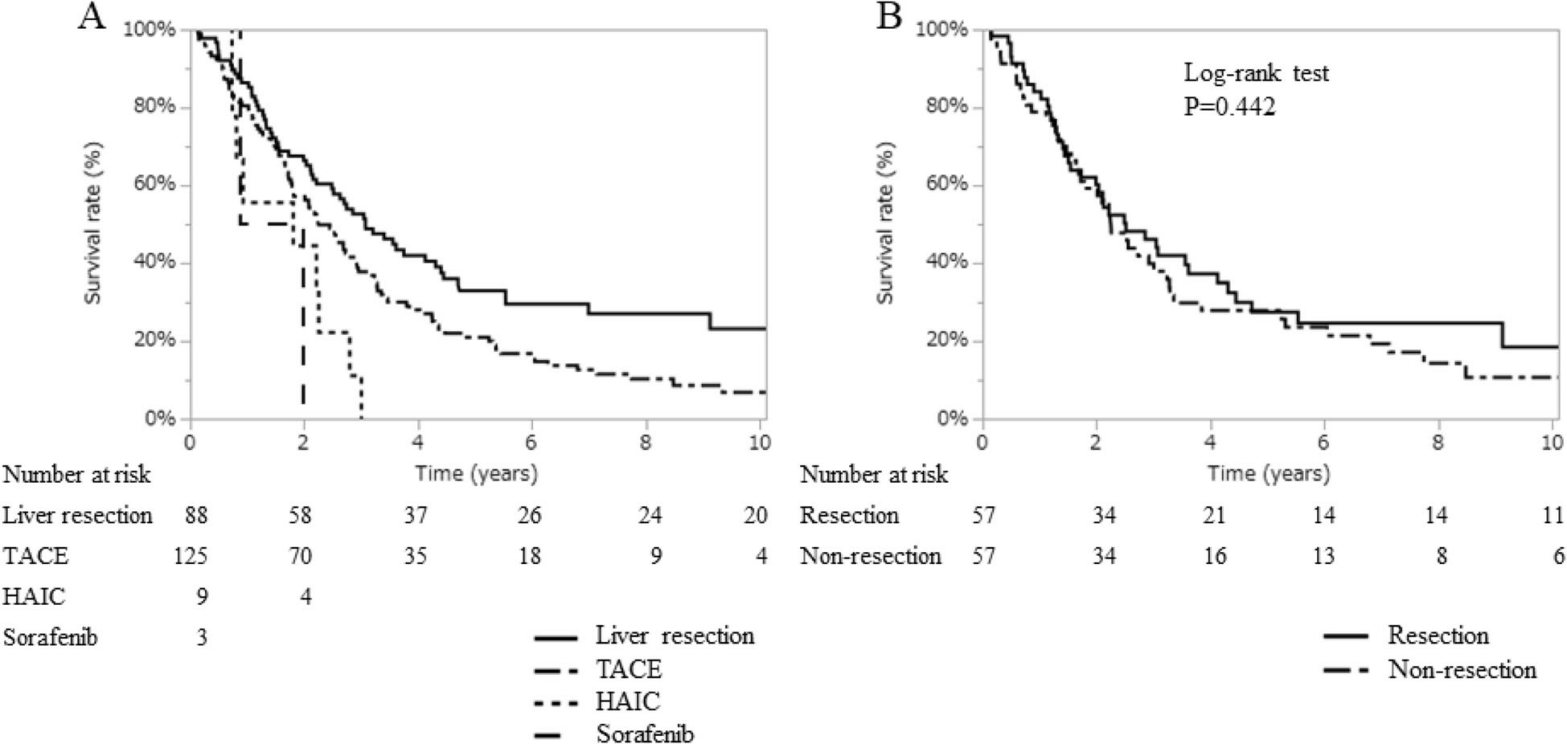
Overall survival in substage B2. **A**. Survival curves showing the prognostic impact by treatment. **B**. Survival curves between resection and non-resection in substage B2 after propensity score matching. HAIC: hepatic arterial infusion chemotherapy, TACE: transarterial chemoembolization

Liver resection also resulted in the highest OS in patients in substage B2 (median OS; 3.1 years, range; 0.2–16.4 years), followed by TACE (median OS: 2.5 years, range: 0.1–12.1 years), HAIC (median OS: 1.8 years, range: 0.7–3.0 years), and sorafenib (median OS: 0.9 years, range: 0.1–2.0 years) (Fig. 3A). Table S5 shows the demographics of patients in substage B2 by treatment types. There was a tendency toward Child-Pugh grade A, more tumors, and larger tumors in the resection group. Univariate and multivariate analysis showed that AFP ≤37 ng/mL, DCP ≤1100 mAU/mL, and liver resection were independent factors for favorable OS (Table S6). Again, patients were divided into resection and non-resection groups. Patient age, platelet count, asparate aminotransferase, ICGR15, Child-Pugh grade, number of tumors, and tumor size significantly differed between these two groups (Table 3). Patients who underwent liver resection were typically younger with better liver function. After PSM, the patient demographics of the two groups were comparable (Table S7), and the OS of the resection and non-resection groups was not significantly different (Fig. 3B). Figure S2B and D showed the distributions of propensity scores before and after matching. The results of the univariate and multivariate analyses of OS are presented in Table S8. Independent factors for favorable OS were AFP ≤37 ng/mL and DCP ≤1100 mAU/mL after PSM, while liver resection was not the independent factor. The CSS for each treatment before PSM is shown in Figure S4A, and CSS in the resection and non-resection groups after PSM is shown in Figure S4B.

**Table 3:** Patient background in substage B2

	Non-resection *N* = 133 (60.2)	Resection *N* = 88 (39.8)	P-value	Cohen’s D
Age (years)	71 (26–93)	69 (31–83)	0.015	0.349
Sex			0.509	0.117
Male	104 (75.9)	71 (80.7)		
Female	33 (24.1)	17 (19.3)		
HBV positive	22 (16.5)	17 (19.3)	0.594	0.065
HCV positive	66 (49.6)	38 (43.2)	0.409	0.115
Plt (×104/mm3)	13.3 (3.7–45.7)	15.7 (5.3–222)	0.049	0.239
PT (%)	85 (14–139)	89 (42–130)	0.244	0.184
T-Bil (mg/dL)	0.8 (0.3–2.2)	0.7 (0.3–2.5)	0.103	0.246
AST (IU/L)	52 (13–432)	47 (17–127)	0.003	0.426
ALT (IU/L)	45 (10–388)	39 (12–174)	0.056	0.243
Alb (g/dL)	3.8 (0.2–4.8)	3.8 (2.5–5.1)	0.929	0.076
ICGR15 (%)	18.1 (1.0–76.2)	15.4 (4.0–79.1)	0.026	0.304
AFP (ng/mL)	31.9 (0.5–191,500)	49.5 (1.7–449,860)	0.134	0.218
DCP (mAU/mL)	301.5 (1.6–197,880)	499.5 (11–529,200)	0.421	0.201
Child-Pugh	110/23 (82.7/17.3)	82/6 (93.2/6.8)	0.026	0.332
A	113 (82.5)	82 (93.2)		
B	24 (17.5)	6 (6.8)		
Tumor number	8 (2–20)	4 (2–20)	< 0.001	0.656
Tumor size (mm)	47 (8–142)	62 (18–355)	< 0.001	0.635

*AFP* alpha-fetoprotein, *Alb* albumin, *AST* asparate aminotransferase, *ALT* alanine aminotransferase, *DCP* des-gamma-carboxyprothorombin, *HBV* hepatitis B virus, *HCV* hepatitis C virus, *ICGR15* indocyanine green retention rate at 15 min, *Plt* platelet count, *PT* prothrombin time, *T. Bil* total bilirubin

### Substage B3

**Fig. 4:**
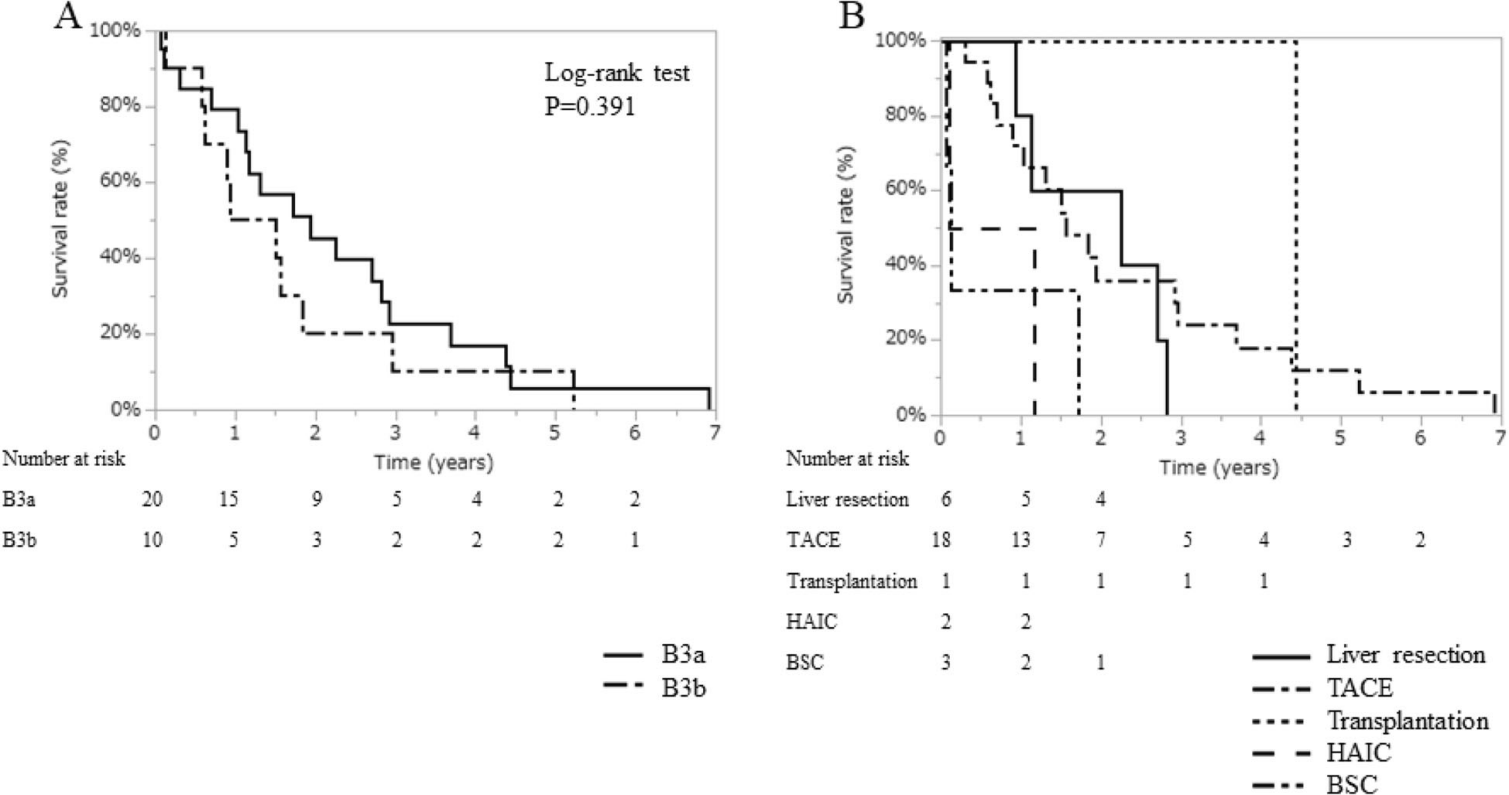
Overall survival in substage B3. **A**. Survival curves between B3a and B3b in substage B3. **B**. Survival curves showing the prognostic impact by treatment. BSC: best supportive care, HAIC: hepatic arterial infusion chemotherapy, TACE: transarterial chemoembolization.

There was no difference in OS between patients in substage B3a and substage B3b (Fig. 4A). Patients in substage B3b had significantly higher DCP than patients in substage B3a, although there was no significant difference in age or liver function. In addition, the number and size of tumors were inherently larger in substage B3b based on the Kinki criteria (Table S9). Liver resection was performed in 5 patients, TACE in 18 patients, HAIC in 2 patients, transplantation in 1 patient, and BSC in 3 patients (Fig. 4B). The CSS between patients in substages B3a and B3b, and for each treatment are shown in Figures S5A, and 5B, respectively.

## Discussion

The Kinki criteria effectively subclassifies patients with BCLC intermediate stage HCC. In substage B1, the resection group in our study had better outcomes than the non-resection group both before and after PSM; in substage B2, the resection group had better outcomes before PSM, but the outcomes after PSM were comparable in the resection and non- resection groups. In this study, we aimed to elucidate the significance of liver resection in HCC patients with BCLC intermediate stage subclasses by comparing the life-prolonging effects of liver resection to those of non-surgical treatment.

The BCLC intermediate stage encompasses a heterogeneous patient population with various levels of liver function and tumor load, requiring further subclassifications for predicting prognosis and treatment recommendations. The proposed treatments in this traditional classification system have been questioned in recent years, as many investigators have reported favorable outcomes from treatment beyond these criteria [14, 15, 24–26]. Yi et al. published a review of BCLC stage B subclassification, mentioning expanded indications for radical treatment as a first option [27]. In recent years, the Kinki criteria have been proposed as a therapeutic strategy model for BCLC intermediate stage HCC [12]. We validated the survival of patients with BCLC intermediate stage HCC according to the Kinki criteria. While a previous report failed to confirm a survival difference using the subclassification proposed by Bolondi et al. [8], our results clearly validated their results. The survival curves of the different subclassifications were stratified with significant differences.

According to the Kinki criteria the recommended treatments for substage B1 include resection, ablation, and TACE. These treatments are all used at our institution. In patients with substage B1, the prognosis was significantly better in patients who underwent liver resection than in patients treated with other modalities; moreover, liver resection was an independent prognostic factor for favorable outcomes. However, the patient demographics were different from those of patients who did not undergo resection. After PSM, the groups were similar in terms of demographics, and the OS of patients who underwent liver resection was still significantly better than that of patients who did not undergo resection. The prognosis for ablation cannot be stated definitively due to the small number of patients who underwent ablation, but the prognosis was better in the resection group, which is consistent with previous reports [14, 28]. Therefore, liver resection should be considered the first choice of treatment for patients in substage B1.

The Kinki criteria treatment recommendations for patients in substage B2 include TACE, HAIC, and sorafenib. We also considered liver resection as a treatment for these patients. Similar to patients in substage B1, the OS of patients in substage B2 who underwent liver resection was significantly better than that of patients in substage B2 who did not undergo resection. In addition, liver resection was also an independent prognostic factor for favorable outcomes in substage B2. However, the OS rates of patients who underwent resection and of those who did not were not significantly different after PSM. The resection group had patients who were younger, had better liver function, and had a milder tumor burden, which could indicate selection bias. The tumors were significantly larger in the resection group. Therefore, in terms of tumor burden, the number of tumors may affect survival more than the tumor size. In substage B2, liver resection did not lead to superior OS; therefore, the choice of treatment should be tailored to the individual patient.

There is a report of prolonged prognosis after liver resection compared with TACE or sorafenib [29], but no significant difference was observed among patients in substage B2. Patients in substage B2 may be considered as having borderline resectable tumors. HAIC has been widely administered in patients with advanced HCC because of its relatively high response rate, prolonged prognosis, and low toxicity [20]. HAIC is an option for patients with multiple lesions in both lobes, as it is difficult to perform TACE in these patients because of liver dysfunction. These is still heterogeneity in tumor condition and liver function in the substages, especially in substage B2. Therefore, a specific treatment should be chosen from the recommended treatment strategies or even beyond these options according to the patient’s condition. The American Association for the Study of Liver Diseases guidelines define TACE as a noncurative treatment that attempts to prolong survival by slowing tumor progression [30], therefore, in resectable substage B2 cases, liver resection may be an option that can provide curative treatment. On the other hand, it has recently been reported that lenvatinib showed a higher objective response rate and longer survival than TACE in intermediate stage patients who do not meet up-to-seven criteria [31]. Furthermore, preserving liver function, one of its strengths, contributes to a better prognosis. It can be expected that lenvatinib may lead to liver resection while maintaining liver function due to the conversion procedure and has implications such as selection of liver resection as neoadjuvant chemotherapy.

According to the Kinki criteria, curative treatments are recommended for patients in substage B3a (including transplantation, ablation, and TACE) while palliative treatments are recommended for treatments in substage B3b (including TACE and HAIC). Patients with substage B3 HCC are typically treated with BSC or palliative care: however, patients within the up-to-7 criteria may undergo TACE or ablation to carefully treat individual HCCs to achieve healing and survival benefits. At our institution, we mainly perform TACE for this purpose. For substage B3 patients with HCC exceeding up-to-7 criteria, our treatment strategy includes HAIC and TACE. The OS was not significantly different between patients in the B3a substage and those in the B3b substage. When possible, liver resection can be performed in these patients, and the results are comparable to other treatments. In addition, the Kinki criteria recommend transplantation for patients with HCC within the up-to-7 criteria; however, the transplantation performed at our hospital was substage B3b. After the downstage, the transplantation was performed and the survival period was 4 years. The number of patients in this study in substage B3 is small. A larger patient population is required in further studies.

There were several limitations to this study. Firstly, the retrospective nature of this study allows for selection and recall biases. We aimed to reduce these biases via PSM. Secondly, there were a relatively small number of cases other than liver resection and TACE; HAIC and sorafenib as treatment methods were not sufficiently studied. Performing HAIC or sorafenib in the BCLC intermediate stage is not the standard of care, which is inevitable. Thirdly, this study was performed at a single center. Lastly, details about the location and depth of the tumors were not recorded. Moreover, multiple tumors located either in a single segment or across segments could not be distinguished. Future studies should include a more thorough description of tumor location.

## Conclusions

The subclassification of patients with BCLC intermediate stage HCC proposed by the Kinki criteria resulted in significant differences in OS among the substages. Patients in substage B1 who underwent liver resection had a significantly better prognosis compared to other treatments. In substages B2 and B3, alternative treatment was required according to the patient’s condition, liver function, and tumor burden.

## Supplementary Information

**Additional file 1: Supplemental figure 1**. Cancer specific survival by substage in BCLC stage B

**Additional file 2: Supplemental figure 2.** Dot plot and histogram (A, C) Before matching. (B, D) After match.

**Additional file 3: Supplemental figure 3** Cancer specific survival in substage B1. A. Survival curves showing the prognostic impact by treatment. B. Survival curves between resection and non-resection in substage B1 after propensity score matching. TACE: transarterial chemoembolization

**Additional file 4: Supplemental figure 4.** Cancer specific survival in substage B2. A. Survival curves showing the prognostic impact by treatment. B. Survival curves between resection and non-resection in substage B2 after propensity score matching. HAIC: hepatic arterial infusion chemotherapy, TACE: transarterial chemoembolization

**Additional file 5: Supplemental figure 5.** Cancer specific survival in substage B3. A. Survival curves between B3a and B3b in substage B3. B. Survival curves showing the prognostic impact by treatment. BSC: best supportive care, HAIC: hepatic arterial infusion chemotherapy, TACE: transarterial chemoembolization.

**Additional file 6.**

## Data Availability

The datasets generated and analyzed for the current study are not publicly available due to institute regulations, but they are available from the corresponding author on reasonable request.

## Abbreviations

AFP: α-fetoprotein
BCLC: Barcelona Clinic Liver Cancer
BSC: Best supportive care
CT: Computed tomography
DCP: Des-γ-carboxy prothrombin
ECOG: Eastern Cooperative Oncology Group
HAIC: Hepatic artery infusion chemotherapy
HCC: Hepatocellular carcinoma
OS: Overall survival; PSM propensity score matching
TACE: Transarterial chemoembolization
