# Supplementary figures and images for: Significance of liver resection for intermediate stage hepatocellular carcinoma according to subclassification
Source: BMC Cancer. 2021 Jun 5;21:668. doi: 10.1186/s12885-021-08421-3 (PMC8180017; doi:10.1186/s12885-021-08421-3)

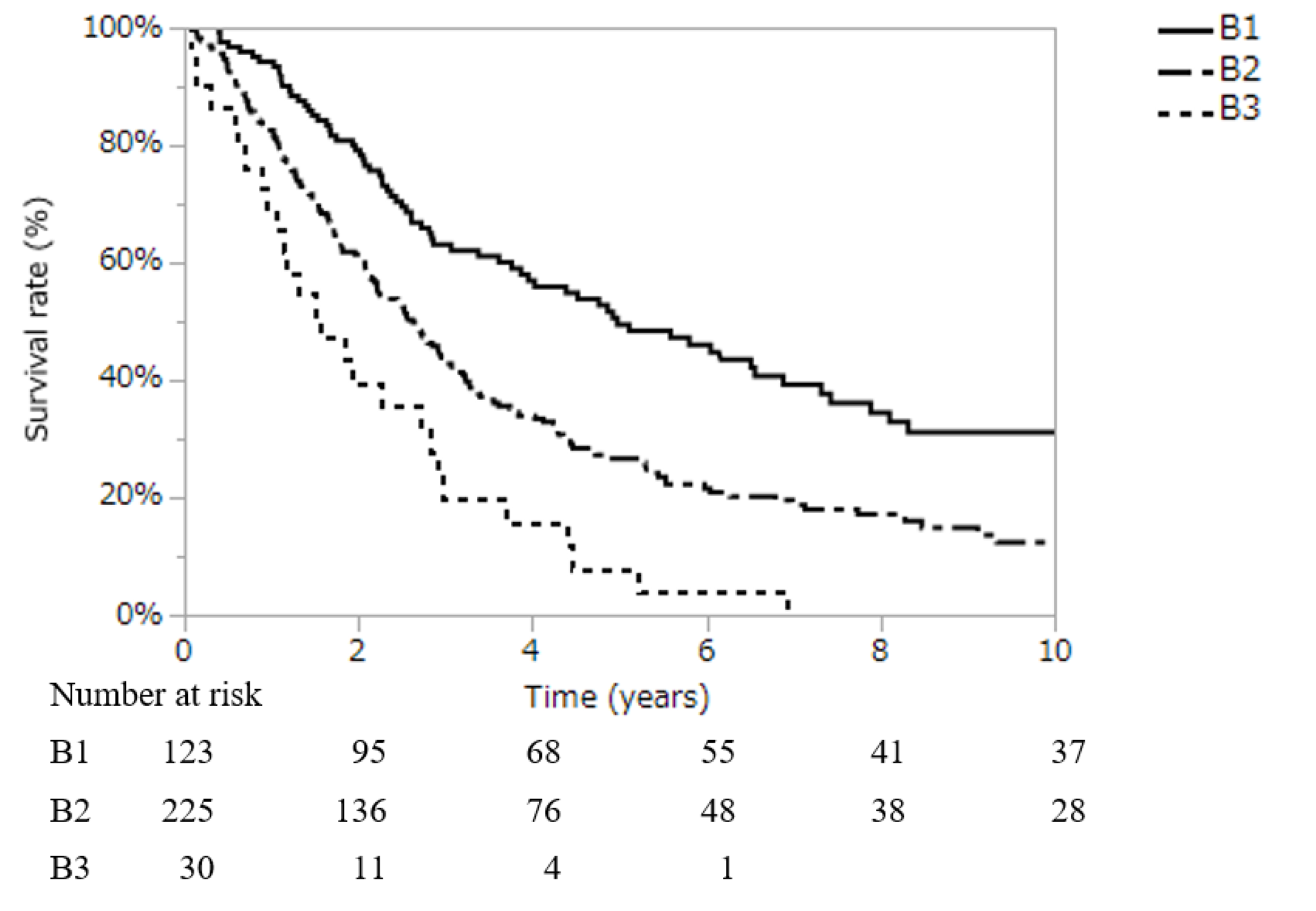

Supplement: Supplementary file 1 — Additional file 1: Supplemental figure 1. Cancer specific survival by substage in BCLC stage B [file 12885_2021_8421_MOESM1_ESM.tif]

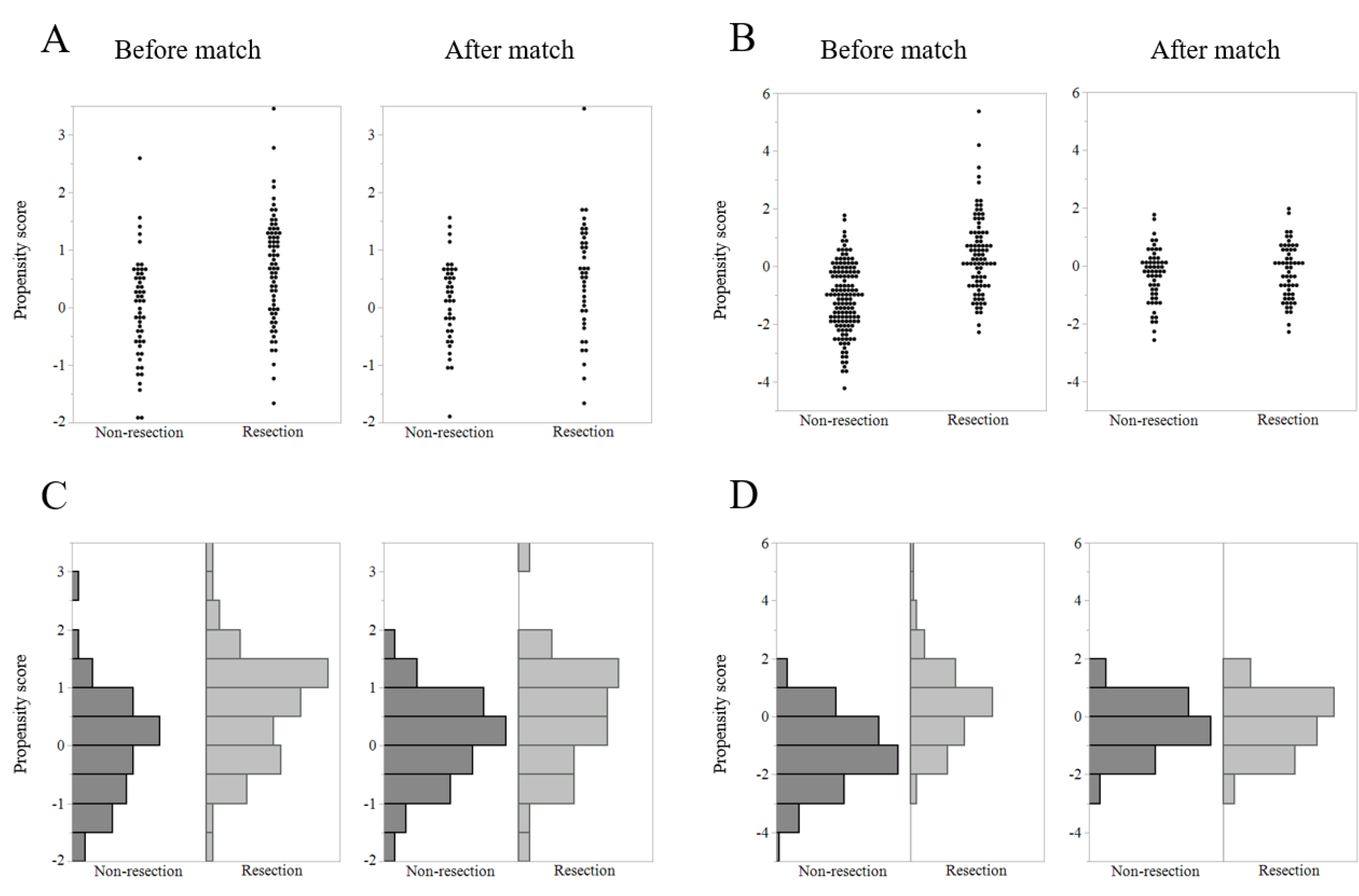

Supplement: Supplementary file 2 — Additional file 2: Supplemental figure 2. Dot plot and histogram (A, C) Before matching. (B, D) After match. [file 12885_2021_8421_MOESM2_ESM.tif]

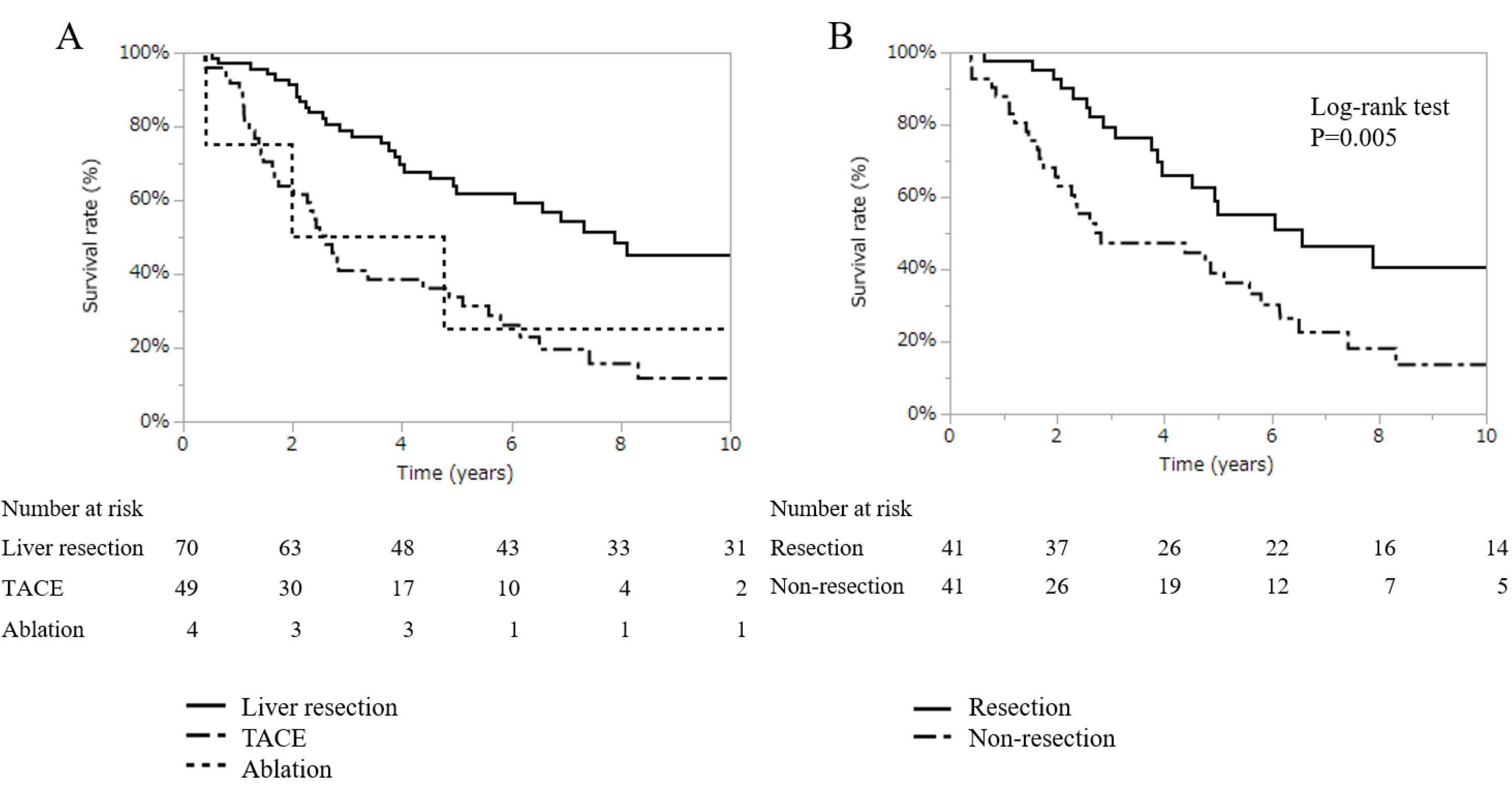

Supplement: Supplementary file 3 — Additional file 3: Supplemental figure 3 Cancer specific survival in substage B1. A. Survival curves showing the prognostic impact by treatment. B. Survival curves between resection and non-resection in substage B1 after propensity score matching. TACE: transarterial chemoembolization [file 12885_2021_8421_MOESM3_ESM.tif]

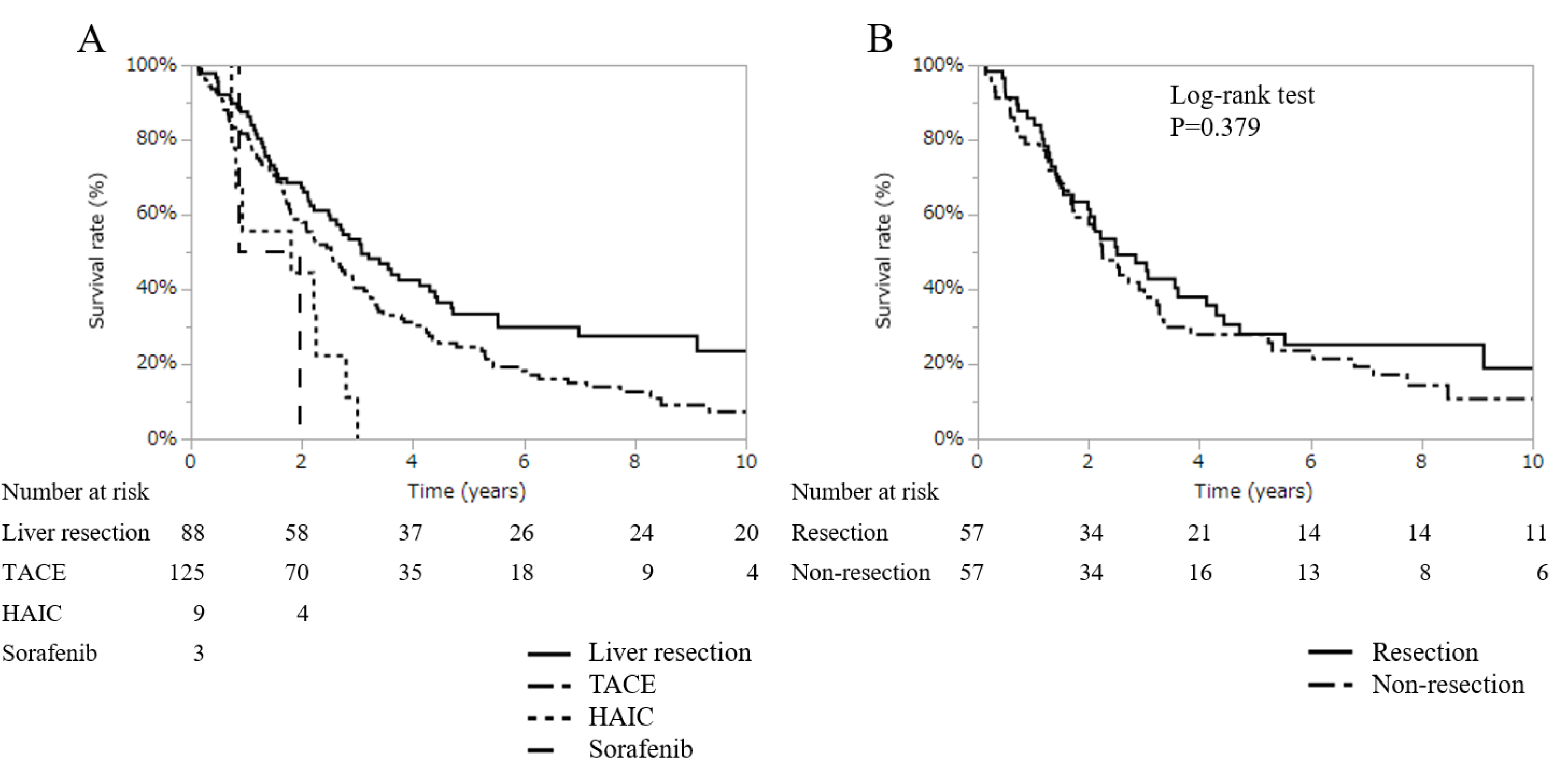

Supplement: Supplementary file 4 — Additional file 4: Supplemental figure 4. Cancer specific survival in substage B2. A. Survival curves showing the prognostic impact by treatment. B. Survival curves between resection and non-resection in substage B2 after propensity score matching. HAIC: hepatic arterial infusion chemotherapy, TACE: transarterial chemoembolization [file 12885_2021_8421_MOESM4_ESM.tif]

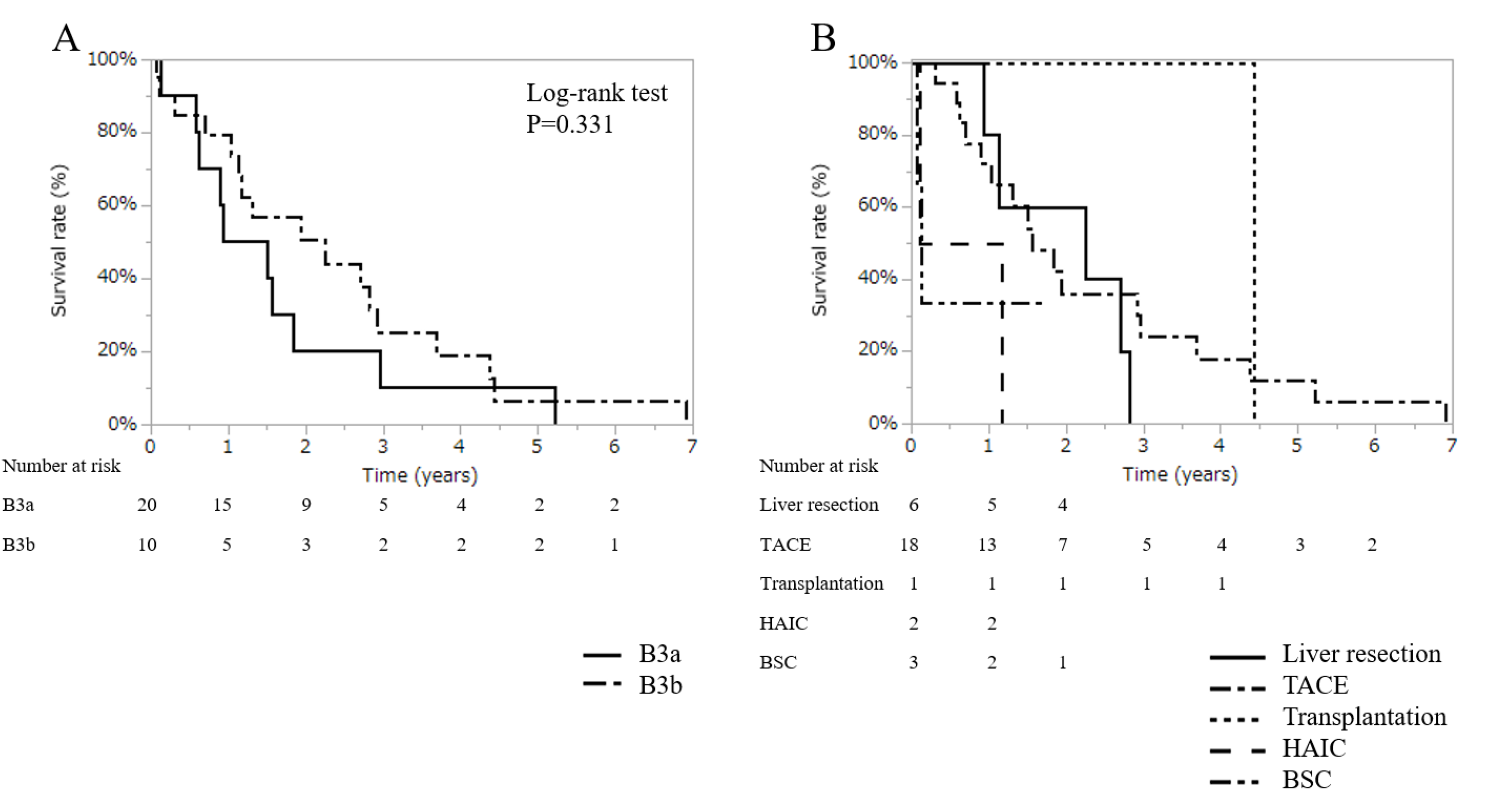

Supplement: Supplementary file 5 — Additional file 5: Supplemental figure 5. Cancer specific survival in substage B3. A. Survival curves between B3a and B3b in substage B3. B. Survival curves showing the prognostic impact by treatment. BSC: best supportive care, HAIC: hepatic arterial infusion chemotherapy, TACE: transarterial chemoembolization. [file 12885_2021_8421_MOESM5_ESM.tif]
