# Supplementary material for: Significance of liver resection for intermediate stage hepatocellular carcinoma according to subclassification
Source: BMC Cancer. 2021 Jun 5;21:668. doi: 10.1186/s12885-021-08421-3 (PMC8180017; doi:10.1186/s12885-021-08421-3)
Supplement: Supplementary file 6 — Additional file 6. [file 12885_2021_8421_MOESM6_ESM.docx]

Supplemental table 1 patient background by treatments in substage B1

|  | TACE  N=49 (39.8) | Ablation  N=4 (3.3) | Resection  N=70 (56.9) |
| --- | --- | --- | --- |
| Age (years) | 71 (51-93) | 70 (58-82) | 68 (45-87) |
| Sex |  |  |  |
| Male | 40 (81.6) | 3 (75.0) | 61 (87.1) |
| Female | 9 (18.4) | 1 (25.0) | 9 (12.9) |
| HBV positive | 5 (10.2) | 1 (25.0) | 10 (14.3) |
| HCV positive | 29 (59.2) | 2 (50.0) | 50 (71.4) |
| Plt (x10^4^/mm^3^) | 10.6 (3.6-25.5) | 7.3 (4.6-9.7) | 12.5 (3.3-189) |
| PT (%) | 83 (36-112) | 74 (64-106) | 84 (24-112) |
| T-Bil (mg/dL) | 0.9 (0.4-2.8) | 0.7 (0.5-1.1) | 0.8 (0.3-2.2) |
| AST (IU/L) | 40 (17-568) | 60 (26-88) | 42 (17-148) |
| ALT (IU/L) | 29 (11-834) | 65 (35-161) | 39 (11-165) |
| Alb (g/dL) | 3.9 (2.8-4.7) | 4.2 (3.5-4.7) | 3.9 (2.9-5.2) |
| ICGR15 (%) | 23.4 (5.5-66.7) | 13.9 (4.0-25.4) | 17.4 (2.6-79.2) |
| AFP (ng/mL) | 25.6 (0.5-4172) | 53.6 (4.6-390) | 14.1 (1.4-8390) |
| DCP (mAU/mL) | 78 (10-17093) | 25 (10-551) | 96 (10-32365) |
| Child-Pugh |  |  |  |
| A | 40 (81.6) | 3 (75.0) | 64 (91.4) |
| B | 9 (18.4) | 1 (25.0) | 6 (8.6) |
| Tumor number | 2 (2-5) | 2 (2-4) | 2 (2-4) |
| Tumor size (mm) | 35 (7-46) | 26 (9-40) | 36 (10-50) |

*AFP* alpha-fetoprotein, *Alb* albumin , *AST* asparate aminotransferase, *ALT* alanine aminotransferase, *DCP* des-gamma-carboxyprothorombin, *HBV* hepatitis B virus, *HCV* hepatitis C virus, *ICGR15* indocyanine green retention rate at 15 min, *Plt* platelet count, *PT* prothrombin time, *TACE* transarterial chemoembolization*T. Bil* total bilirubin

Supplemental table 2 Univariate and multivariate analysis of risk factors for OS in substage B1

|  | Univariate analysis | | | Multivariate analysis | | |
| --- | --- | --- | --- | --- | --- | --- |
|  | P value | HR | 95%CI | P value | HR | 95%CI |
| Age >70 (years) | 0.095 | 1.484 | 0.932-2.349 |  |  |  |
| Sex Male | 0.209 | 1.474 | 0.792-2.554 |  |  |  |
| HBV positive | 0.192 | 1.555 | 0.814-3.359 |  |  |  |
| HCV positive | 0.379 | 1.247 | 0.768-2.092 |  |  |  |
| ICGR15 >23.8 (%) | 0.015 | 1.842 | 1.121-3.152 | 0.071 | 1.633 | 0.961-2.884 |
| AFP >37 (ng/mL) | 0.017 | 1.761 | 1.108-2.778 | 0.014 | 1.816 | 1.128-2.908 |
| DCP >1100 (mAU/mL) | 0.408 | 1.287 | 0.689-2.239 |  |  |  |
| Child-Pugh B | 0.009 | 2.364 | 1.261-4.148 | 0.161 | 1.601 | 0.821-2.951 |
| Tumor number >5 | 0.029 | 1.768 | 1.062-2.867 | 0.291 | 1.326 | 0.778-2.204 |
| Tumor size >50 (mm) | 0.608 | 1.373 | 0.335-3.692 |  |  |  |
| Treatment other than resection | <0.001 | 2.787 | 1.749-4.489 | <0.001 | 2.451 | 1.499-4.043 |

*AFP* alpha-fetoprotein, *DCP* des-gamma-carboxyprothorombin, *HBV* hepatitis B virus, *HCV* hepatitis C virus, *ICGR15* indocyanine green retention rate at 15 min

Supplemental table 3 Patient background in substage B1 after propensity score match

|  | Non-resection  N=41 (50.0) | Resection  N=41 (50.0) | P-value | Cohen’s D |
| --- | --- | --- | --- | --- |
| Age (years) | 70 (47-87) | 71 (69-85) | 0.317 | 0.057 |
| Sex |  |  | 0.349 | 0.276 |
| Male | 33 (80.5) | 37 (90.2) |  |  |
| Female | 8 (19.5) | 4 (9.8) |  |  |
| HBV positive | 6 (14.6) | 3 (7.3) | 0.482 | 0.235 |
| HCV positive | 25 (61.0) | 31 (75.6) | 0.235 | 0.317 |
| Plt (x104/mm3) | 12.9 (4.2-189) | 12.2 (8.1-212) | 0.924 | 0.217 |
| PT (%) | 84 (66-108) | 82 (33-83) | 0.127 | 0.061 |
| T-Bil (mg/dL) | 0.9 (0.4-1.5) | 0.8 (0.6-0.9) | 0.388 | 0.037 |
| AST (IU/L) | 41 (20-141) | 55 (25-148) | 0.378 | 0.099 |
| ALT (IU/L) | 39 (12-165) | 66 (20-99) | 0.366 | 0.026 |
| Alb (g/dL) | 3.8 (2.9-5.2) | 3.8 (3.6-4.5) | 0.737 | 0.076 |
| ICGR15 (%) | 19.8 (2.6-79.2) | 20.7 (6.9-27.5) | 0.962 | 0.041 |
| AFP (ng/mL) | 12.4 (1.4-720) | 35.9 (2.3-1197) | 0.555 | 0.196 |
| DCP (mAU/mL) | 106 (10-4788) | 597 (24-16123) | 0.198 | 0.121 |
| Child-Pugh | 35/6 (85.4/14.6) | 37/4 (90.2/9.8) | 0.737 | 0.147 |
| A | 35 (85.4) | 37 (90.2) |  |  |
| B | 6 (14.6) | 4 (9.8) |  |  |
| Tumor number | 2 (2-4) | 2 (2-3) | 0.362 | 0.122 |
| Tumor size (mm) | 35 (12-50) | 41 (36-45) | 0.273 | 0.161 |

*AFP* alpha-fetoprotein, *Alb* albumin , *AST* asparate aminotransferase, *ALT* alanine aminotransferase, *DCP* des-gamma-carboxyprothorombin, *HBV* hepatitis B virus, *HCV* hepatitis C virus, *ICGR15* indocyanine green retention rate at 15 min, *Plt* platelet count, *PT* prothrombin time, *T. Bil* total bilirubin

Supplemental table 4 Univariate and multivariate analysis of risk factors for OS in substage B1 after propensity score match

|  | Univariate analysis | | | Multivariate analysis | | |
| --- | --- | --- | --- | --- | --- | --- |
|  | P value | HR | 95%CI | P value | HR | 95%CI |
| Age >70 (years) | 0.688 | 1.121 | 0.635-1.947 |  |  |  |
| Sex Male | 0.398 | 1.469 | 0.549-1.462 |  |  |  |
| HBV positive | 0.245 | 1.679 | 0.724-4.891 |  |  |  |
| HCV positive | 0.804 | 1.078 | 0.604-2.027 |  |  |  |
| ICGR15 >23.8 (%) | 0.134 | 1.574 | 0.875-3.004 |  |  |  |
| AFP >37 (ng/mL) | <0.001 | 3.074 | 1.748-5.429 | <0.001 | 2.897 | 1.641-5.137 |
| DCP >1100 (mAU/mL) | 0.951 | 1.022 | 0.532-2.166 |  |  |  |
| Child-Pugh B | 0.103 | 1.984 | 0.859-4.023 |  |  |  |
| Tumor number >5 | 0.041 | 1.698 | 1.235-3.285 | 0.188 | 1.494 | 0.816-2.651 |
| Treatment other than resection | 0.006 | 2.167 | 1.243-3.871 | 0.011 | 2.071 | 1.175-3.752 |

*AFP* alpha-fetoprotein, *DCP* des-gamma-carboxyprothorombin, *HBV* hepatitis B virus, *HCV* hepatitis C virus, *ICGR15* indocyanine green retention rate at 15 min

Supplemental table 5 patient background by treatments in substage B2

|  | TACE  N=125 (55.6) | HAIC  N=9 (4.0) | Systemic chemotherapy  N=4 (1.3) | Liver resection  N=88 (39.1) |
| --- | --- | --- | --- | --- |
| Age (years) | 71 (26-93) | 70 (49-86) | 69 (68-75) | 69 (31-83) |
| Sex |  |  |  |  |
| Male | 94 (75.2) | 7 (77.8) | 3 (100) | 71 (80.7) |
| Female | 31 (24.8) | 2 (22.2) | 0 (0) | 17 (19.3) |
| HBV positive | 20 (16.0) | 3 (33.3) | 0 (0) | 17 (19.3) |
| HCV positive | 59 (47.2) | 7 (77.8) | 1 (33.3) | 38 (43.2) |
| Plt (x104/mm3) | 13.3 (3.7-99) | 16.0 (7.3-32.5) | 12.7 (7.4-28.2) | 15.7 (5.3-222) |
| PT (%) | 85 (14-119) | 87 (68-139) | 89 (76-99) | 89 (42-130) |
| T-Bil (mg/dL) | 0.8 (0.3-2.2) | 1.1 (0.4-1.7) | 0.7 (0.5-0.9) | 0.7 (0.3-2.5) |
| AST (IU/L) | 50 (13-432) | 71 (50-97) | 69 (37-75) | 47 (17-127) |
| ALT (IU/L) | 44 (10-388) | 57 (21-92) | 48 (18-87) | 39 (12-174) |
| Alb (g/dL) | 3.9 (2.0-4.8) | 3.7 (3.0-4.8) | 3.4 (3.1-3.6) | 3.8 (2.5-5.1) |
| ICGR15 (%) | 17.7 (1.0-76.2) | 20.0 (6.3-49.2) | 20.8 (14.5-27.2) | 15.4 (4.0-79.1) |
| AFP (ng/mL) | 17.7 (0.5-129460) | 131 (12.6-191500) | 1205 (7.8-1271) | 49.5 (1.7-449860) |
| DCP (mAU/mL) | 1616 (1.6-197880) | 1619 (52-39781) | 232 (134-5412) | 499.5 (11-529200) |
| Child-Pugh | 103/22 (82.4/17.6) | 7/2 (77.8/22.2) | 3/0 (100/0) | 82/6 (93.2/6.8) |
| A | 103 (82.4) | 7 (77.8) | 3 (100) | 82 (93.2) |
| B | 22 (17.6) | 2 (22.2) | 0 (0) | 6 (6.8) |
| Tumor number | 8 (2-10) | 10 (4-20) | 5 (3-8) | 4 (2-20) |
| Tumor size (mm) | 47 (8-142) | 40 (23-100) | 30 (27-95) | 62 (18-355) |

*AFP* alpha-fetoprotein, *Alb* albumin , *AST* asparate aminotransferase, *ALT* alanine aminotransferase, *DCP* des-gamma-carboxyprothorombin, *HAIC* hepatic arterial infusion chemotherapy, *HBV* hepatitis B virus, *HCV* hepatitis C virus, *ICGR15* indocyanine green retention rate at 15 min, *Plt* platelet count, *PT* prothrombin time, *TACE* transarterial chemoembolization, *T. Bil* total bilirubin

Supplemental table 6 Univariate and multivariate analysis of risk factors for OS in substage B2

|  | Univariate analysis | | | Multivariate analysis | | |
| --- | --- | --- | --- | --- | --- | --- |
|  | P value | HR | 95%CI | P value | HR | 95%CI |
| Age >70 (years) | 0.722 | 1.056 | 0.779-1.429 |  |  |  |
| Sex Male | 0.222 | 1.251 | 0.869-1.761 |  |  |  |
| HBV positive | 0.665 | 1.088 | 0.732-1.571 |  |  |  |
| HCV positive | 0.898 | 0.981 | 0.726-1.323 |  |  |  |
| ICGR15 >30.0 (%) | 0.088 | 1.403 | 0.947-2.021 |  |  |  |
| AFP >37 (ng/mL) | <0.001 | 1.896 | 1.402-2.572 | <0.001 | 1.966 | 1.446-2.681 |
| DCP >1100 (mAU/mL) | <0.001 | 1.762 | 1.291-2.391 | <0.001 | 1.903 | 1.379-2.611 |
| Child-Pugh B | 0.082 | 1.495 | 0.946-2.258 |  |  |  |
| Tumor number >5 | 0.467 | 1.135 | 0.811-1.621 |  |  |  |
| Tumor size >50 (mm) | 0.164 | 1.236 | 0.916-1.669 |  |  |  |
| Treatment other than resection | 0.009 | 1.506 | 1.102-2.079 | <0.001 | 1.946 | 1.405-2.725 |

*AFP* alpha-fetoprotein, *DCP* des-gamma-carboxyprothorombin, *HBV* hepatitis B virus, *HCV* hepatitis C virus, *ICGR15* indocyanine green retention rate at 15 min

Supplemental table 7 Patient background in substage B2 after propensity score match

|  | Non-resection  N=57 (50.0) | Resection  N=57 (50.0) | P-value | Cohen’s D |
| --- | --- | --- | --- | --- |
| Age (years) | 67 (43-85) | 70 (42-82) | 0.464 | 0.085 |
| Sex |  |  | 1 | 0.045 |
| Male | 48 (84.2) | 47 (82.5) |  |  |
| Female | 9 (15.8) | 10 (17.5) |  |  |
| HBV positive | 17 (29.8) | 8 (14.1) | 0.168 | 0.386 |
| HCV positive | 24 (42.1) | 31 (54.4) | 0.261 | 0.248 |
| Plt (x104/mm3) | 13.3 (3.7-34.1) | 13.2 (5.3-122) | 0.624 | 0.032 |
| PT (%) | 86 (14-119) | 89 (42-130) | 0.912 | 0.012 |
| T-Bil (mg/dL) | 0.7 (0.3-1.7) | 0.8 (0.3-1.9) | 0.956 | 0.027 |
| AST (IU/L) | 49 (20-137) | 52 (17-127) | 0.957 | 0.037 |
| ALT (IU/L) | 44 (15-128) | 41 (12-163) | 0.501 | 0.067 |
| Alb (g/dL) | 3.9 (2.8-4.8) | 3.8 (2.8-5.1) | 0.524 | 0.063 |
| ICGR15 (%) | 17.5 (2-76.2) | 16.2 (4-79.1) | 0.883 | 0.022 |
| AFP (ng/mL) | 31.3 (0.5-129460) | 54.9 (2-23800) | 0.291 | 0.122 |
| DCP (mAU/mL) | 684 (1.6-167320) | 247 (13-529200) | 0.249 | 0.022 |
| Child-Pugh |  |  | 1 | 0 |
| A | 52 (91.2) | 52 (91.2) |  |  |
| B | 5 (8.8) | 5 (8.8) |  |  |
| Tumor number | 4 (2-10) | 4 (2-20) | 0.394 | 0.105 |
| Tumor size (mm) | 55 (22-142) | 50 (18-190) | 0.223 | 0.075 |

*AFP* alpha-fetoprotein, *Alb* albumin , *AST* asparate aminotransferase, *ALT* alanine aminotransferase, *DCP* des-gamma-carboxyprothorombin, *HBV* hepatitis B virus, *HCV* hepatitis C virus, *ICGR15* indocyanine green retention rate at 15 min, *Plt* platelet count, *PT* prothrombin time, *T. Bil* total bilirubin

Supplemental table 8 Univariate and multivariate analysis of risk factors for OS in substage B2 after propensity score match

|  | Univariate analysis | | | Multivariate analysis | | |
| --- | --- | --- | --- | --- | --- | --- |
|  | P value | HR | 95%CI | P value | HR | 95%CI |
| Age >70 (years) | 0.134 | 1.401 | 0.903-2.214 |  |  |  |
| Sex Male | 0.763 | 1.094 | 0.627-2.068 |  |  |  |
| HBV positive | 0.129 | 1.476 | 0.887-2.367 |  |  |  |
| HCV positive | 0.211 | 1.314 | 0.856-2.029 |  |  |  |
| ICGR15 >30.0 (%) | 0.294 | 1.393 | 0.733-2.444 |  |  |  |
| AFP >37 (ng/mL) | 0.006 | 1.822 | 1.181-2.833 | 0.011 | 1.773 | 1.135-2.791 |
| DCP >1100 (mAU/mL) | <0.001 | 2.428 | 1.555-3.764 | 0.004 | 2.021 | 1.255-3.243 |
| Child-Pugh B | 0.839 | 1.091 | 0.423-2.302 |  |  |  |
| Tumor number >5 | 0.978 | 1.006 | 0.643-1.615 |  |  |  |
| Tumor size >50 (mm) | 0.019 | 1.667 | 1.084-2.599 | 0.154 | 1.407 | 0.881-2.266 |
| Treatment other than resection | 0.443 | 1.181 | 0.772-1.819 |  |  |  |

*AFP* alpha-fetoprotein, *DCP* des-gamma-carboxyprothorombin, *HBV* hepatitis B virus, *HCV* hepatitis C virus, *ICGR15* indocyanine green retention rate at 15 min

Supplemental table 9 patient background in substage B3

|  | B3a (N=10) | B3b (N=19) | P-value |
| --- | --- | --- | --- |
| Age (years) | 68 (50-86) | 68 (53-85) | 0.582 |
| Sex | 6/4 (60/40) | 15/4 (78.9/2) | 0.391 |
| Male | 6 (60) | 15 (78.9) |  |
| Female | 4 (40) | 4 (22.1) |  |
| HBV positive | 2 (22.2) | 4 (21.1) | 1 |
| HCV positive | 6 (75) | 6 (31.6) | 0.087 |
| Plt (x104/mm3) | 9.3 (5.3-14.1) | 11.3 (4.9-94.3) | 0.152 |
| PT (%) | 66 (37-93) | 54 (55-101) | 0.877 |
| T-Bil (mg/dL) | 1.0 (0.3-2.1) | 1.7 (0.3-3.2) | 0.067 |
| AST (IU/L) | 44 (32-65) | 71 (16-296) | 0.108 |
| ALT (IU/L) | 31 (20-70) | 41 (11-204) | 0.388 |
| Alb (g/dL) | 2.8 (2-3.3) | 2.7 (2.2-3.5) | 0.824 |
| ICGR15 (%) | 43.7 (18.2-69.2) | 36.9 (6.6-76.9) | 0.445 |
| Child Pugh score | 8 (8-9) | 8 (8-9) | 1 |
| AFP (ng/mL) | 41.8 (8.5-10750) | 60.8 (0.5-25910) | 0.679 |
| DCP (mAU/mL) | 116 (10-2070) | 1122 (24-4673500) | 0.027 |
| Child-Pugh score 8/9 | 7/3 (70.0/30.0) | 13/6 (68.4/31.6) | 1 |
| Tumor number | 2 (2-4) | 4 (2-10) | 0.016 |
| Tumor size (mm) | 33 (15-50) | 54 (13-200) | 0.002 |
| Hx/TACE/BSC | 1/8/1 (10.0/80.0/10.0) | 5/12/2 (26.3/63.2/10.5) | 0.572 |

*AFP* alpha-fetoprotein, *Alb* albumin , *AST* asparate aminotransferase, *ALT* alanine aminotransferase, *DCP* des-gamma-carboxyprothorombin, *HBV* hepatitis B virus, *HCV* hepatitis C virus, *ICGR15* indocyanine green retention rate at 15 min, *Plt* platelet count, *PT* prothrombin time, *T. Bil* total bilirubin
